# Diverse perspectives on social determinants of multicultural adolescents: A focus group study

**DOI:** 10.1093/eurpub/ckac131.525

**Published:** 2022-10-25

**Authors:** H Lee, H Lee, M Lee, Y Kim, S Kim, J Lee, SY Shim, J Seo

**Affiliations:** 1 College of Nursing, Yonsei University, Seoul, South Korea; 2 Brain Korea 21 FOUR Project, Yonsei University, Seoul, South Korea; 3 Mo-Im Kim Nursing Research Institute, Yonsei University, Seoul, South Korea; 4 College of Nursing, Kosin University, Busan, South Korea; 5 School of Nursing, Soonchunhyang University, Cheonan, South Korea

## Abstract

**Background:**

As Korea transforms into a multicultural society, social vulnerability of the multicultural adolescents (MAs) puts them at risk for poor health and health disparities. However, there is shortage of evidence on social determinants of health (SDH), which refers to the circumstances of people from birth to death, which affects their health outcomes, for MAs. Thus, this study aims to explore the SDH of MAs from diverse stakeholders’ perspectives.

**Methods:**

This qualitative study comprised 17 focus group interviews with 99 participants (MAs, peers, parents, teachers, neighbors, and community leaders), conducted from June to September 2020. The directed content analysis was conducted using the Minority Health and Health Disparities Research Framework with a high rigor level based on the four criteria of Lincoln and Guba’s trustworthiness.

**Results:**

The participants addressed SDH of MAs in five domains: biological (vulnerability and mechanism); behavioral (health behavior, family/school/peer functioning, and policies and laws); physical/built environment (school/community environment); sociocultural environment (MAs'/parental sociodemographic, language proficiency, MAs'/parental acculturation, social network, and response to/interpersonal/local/societal structural discrimination); and health care system (insurance coverage, MAs'/parental health literacy, availability of services, and health care policies). However, limited SDH in biological and physical/built environment domains were identified.

**Conclusions:**

SDH in sociocultural environment domain and interpersonal influence level were the most commonly addressed. Interpersonal discrimination of both MAs and parents were found to be the most important SDH. These findings suggest that future programs to enhance MAs’ health may be targeted toward reducing discrimination and involving their parents.

**Key messages:**

• Sociocultural environment was found to be the most salient SDH domain to affect MAs’ health.

• SDH of MAs are linked to discrimination and their parents’ sociocultural aspects such as acculturation.

